# Mdig promotes oncogenic gene expression through antagonizing repressive histone methylation markers

**DOI:** 10.7150/thno.36220

**Published:** 2020-01-01

**Authors:** Qian Zhang, Chitra Thakur, Yao Fu, Zhuoyue Bi, Priya Wadgaonkar, Liping Xu, Zhipeng Liu, Wanqing Liu, Jian Wang, Benjamin L. Kidder, Fei Chen

**Affiliations:** 1 Department of Pharmaceutical Sciences, Eugene Applebaum College of Pharmacy and Health Sciences, Wayne State University, 259 Mack Avenue, Detroit, MI 48201, USA;; 2 Department of Medicinal Chemistry and Molecular Pharmacology, Purdue University, 575 W. Stadium Avenue, West Lafayette, IN 47907, USA;; 3 Department of Pharmacology, School of Medicine, Wayne State University, 540 E. Canfield Street, Detroit, MI 48201, USA;; 4 Department of Pathology, School of Medicine, Wayne State University, 540 E. Canfield Street, Detroit, MI 48201, USA;; 5 Department of Oncology and the Karmanos Cancer Institute, School of Medicine, Wayne State University, 4100 John R Street, Detroit, MI 48201, USA.

**Keywords:** Mdig, CRISPR-Cas9, H3K9me3, Cancer stemness, Inflammation.

## Abstract

The mineral dust-induced gene (mdig) is overexpressed in a number of human cancers, suggesting critical roles of this gene played on the pathogenesis of cancers. Unlike several other JmjC-domain containing proteins that exhibit histone demethylase activity, it remains enigmatic whether mdig is involved in the demethylation processes of the histone proteins.

**Methods:** To provide direct evidence suggesting contribution of mdig to the demethylation of histone proteins, we recently examined the histone methylation profiles in human bronchial epithelial cells as well as two cancer cell lines with mdig knockout through CRISPR-Cas9 gene editing.

**Results:** Global histone methylation analysis revealed a pronounced increase of the repressive histone trimethylation in three different cell types with mdig depletion, including trimethylation of lysines 9 and 27 on histone H3 (H3K9me3, H3K27me3) and trimethylation of lysine 20 of histone H4 (H4K20me3). Importantly, data from both ChIP-seq and RNA-seq suggested that genetic disruption of mdig enriches repressive histone trimethylation and inhibits expression of target genes in the oncogenic pathways of cell growth, stemness of the cells, tissue fibrosis, and cell motility.

**Conclusion:** Taken together, our study provides the first insight into the molecular effects of mdig as an antagonist for repressive histone methylation markers and suggests that targeting mdig may represent a new area to explore in cancer therapy.

## Introduction

The mineral dust-induced gene (mdig) was first identified from alveolar macrophages of the people who were working in the coal mining industry 1, 2. The same gene was also independently discovered in human glioblastoma cell line T98G engineered with ectopic c-myc overexpression and named as myc-induced nuclear antigen 53 (mina53) 3. In addition, mdig was also characterized as a nucleolar protein NO52 in Xenopus laevis oocytes 4. The mdig protein contains a conserved Jumonji C (JmjC) domain, the signature motif of the histone demethylase family. Indeed, some *in vitro* studies suggested that mdig is able to reduce the level of histone H3 lysine 9 trimethylation (H3K9me3) in cells from lung cancer 5, glioblastoma 6, 7 and hepatocellular carcinoma 8. However, structure-functional tests of this protein clearly indicated that mdig is a protein hydroxylase for histidine-39 of ribosomal protein L27a, rather than a histone demethylase, and accordingly, was re-named ribosome oxygenase 2 (RIOX2) 9, 10.

In ectopic expression using human lung cancer cell line A549, we had previously demonstrated that overexpression of mdig not only diminished the overall heterochromatin conformation of the cells, but also restored expression of H19, an imprinted gene for a long non-coding RNA (lncRNA) 5. Using chromatin immunoprecipitation and polymerase chain reaction (ChIP-PCR) approach, we noted that enforced overexpression of mdig was capable of down-regulating H3K9me3 in the imprinting control region (ICR) of the H19-IGF2 gene loci 5. Meanwhile, histone demethylation assay *in vitro* showed some detectable demethylase activity of the immunoprecipitation-enriched mdig protein toward the lysine 9 trimethylated histone H3 peptide. As the one of the first reported lncRNAs, emerging evidence suggested an increased expression and oncogenic activity of H19 in most of the human tumors 11, 12. More strikingly, exosomes from the carcinoma-associated fibroblasts contain high level of H19 and are highly capable of enhancing the expression of the stemness genes of the colorectal cancer stem cells (CSCs) 13. Furthermore, H19 plays pivotal roles on the induction of fibrosis of the liver, kidney and lung in response to bile duct ligation, TGFβ and bleomycin, respectively 14-16.

The oncogenic property of mdig is supported by the fact that many human cancers, including cancers in colon, esophagus, lung, gingival, lymphocytes, kidney, neural system, liver, breast, pancreas, stomach, etc, exhibited an elevated expression of mdig 2. This notion is in agreement with findings that mdig promotes cell proliferation, cell cycle transition, or anti-apoptotic responses in several cell types 1, 17. In datasets of cancer patient overall or progress free survival, there is a clear association between higher mdig expression and poorer survival of the cancer patients, with few exceptions. In lung cancer, the prognostic indication of mdig is largely depending on the histological subtypes and/or cancer stages, e.g., higher mdig predicts poorer first progression survival of the lung adenocarcinomas, but not the squamous cell carcinoma 17, 18. Similarly, higher expression of mdig predicts poorer survival of the breast cancer patients without lymph node metastasis 19. Among the breast cancer patients with lymph node metastasis, in contrast, the higher level of mdig indicated a much better survival 20.

The mice with heterozygous knockout of mdig gene are developmentally normal, or even have longer life span than the wild-type mice from the same progenies 21. In response to pharyngeal aspiration-based silica challenge, the knockout mice showed a significant alleviation of lung fibrosis, along with a pronounced reduction of T helper 17 (Th17) cell infiltrated into the lung interstitium, implying that mdig is a prominent contributing factor to lung fibrosis and inflammation. Genetic disruption of the mdig genes, therefore, may ameliorate the basal and environmental hazard-induced inflammatory responses, leading to a generally healthy or improved outcome. However, there are numerous genes and proteins involved in the initiation, progression, resolution, and repair of inflammation in any given tissue or organ. For the inflammatory Th17 cells, many regulatory factors determine the lineage polarization, functional specialization and localization to the inflammatory sites of the Th17 cells 22. It is unclear at the present how mdig plays its roles on inflammation or the inflammatory Th17 cells.

To better understand the function of mdig in inflammation and cancer, and clarify whether mdig is involved in the demethylation of histone proteins, in the present report we employed a CRISPR-Cas9 gene editing technique to knockout mdig in human bronchial epithelial cells, breast cancer cells and lung cancer cells, and provided evidence showing that mdig antagonizes the common repressive histone trimethylation markers 23, including H3K9me3, H3K27me3 and H4K20me3 in these three different cell types. In contrast, mdig is unable to demethylate H3K4me3, H3K36me3, or asymmetric dimethylation of arginine 8 of histone H3 (H3R8me2a). Knockout of mdig, therefore, resulted in a substantial enrichment of these repressive histone trimethylation markers on the gene loci involved in cell growth, stemness, inflammation, and cell migration.

## Materials and Methods

### Construction of the CRISPR-Cas9 vector

To generate the CRISPR-Cas9 plasmid, mdig CDS sequence was inputted into the CRISPR Design tool (http://crispr.mit.edu/), and single guide RNA (sgRNA) sequence targeting on exon 3 of mdig was selected. The sense and antisense primer sequences are 5'-CACCGAATGTGTACATAACTCCCGC-3' and 5'-AAACGCGGGAGTTATGTACACATTC-3', respectively. Single-stranded sense and antisense primers were annealed to form double-strand oligos in 95 °C for 5 min, and then cooled down to room temperature at a speed of 5°C/min. Vector pSpCas9-2A-Blast was digested with BpiI (BbsI) restriction enzymes (Thermo Fisher Scientific, Ann Arbor, MI). sgRNA pairs and linearized vector were ligated by T4 DNA ligase (Thermo Fisher Scientific) for 10 min at 22°C. Then the ligation product was transferred into DH5α competent *E.coli* strain (Thermo fisher scientific) according to the manufacturer's protocol.

### Transfection and colonies selection

BEAS-2B cells, A549 lung cancer cell line, and triple negative breast cancer cell line MDA-MB-231 were purchased from ATCC (Manassas, VA). The cells were seeded in 6-well plates at a number of 2.5 × 10^5^/well and transfected with the constructed CRISPR-Cas9 vector by Lipofectamine 2000 (Thermo Fisher Scientific) according to the manufacture's protocol. Forty eight hours after transfection, cells were sub-cultured in 10 cm dish for 24h, followed by 2μg/ml of Blasticidin (Thermo Fisher Scientific) selection for 2 weeks. Cell colonies were collected for screening of mdig expression by Western blotting. Colonies without mdig knockout were used as wild type cells (WT), whereas colonies with successful mdig knockout were designated as knockout (KO) cells.

### Silencing of mdig by siRNAs and mdig rescue experiment

Control siRNA and mdig siRNAs were purchased from Qiagen (Valencia, CA). pCMV6-AC-GFP (PS100010) and pCMV6-AC-mdig-GFP (RG214829) were purchased from Origene (Rockville, MD). BEAS-2B cells, 3 × 10^5^/well were seeded in 6-well plate, and grown to 70% confluency. siRNAs and plasmid DNA were transfected into cells using Lipofectamine RNAiMAX (Thermo Fisher Scientific) and Lipofectamine 2000 (Thermo Fisher Scientific), respectively, according to manufacturer's protocol. For rescue experiment, co-transfection of siRNA and mdig expression plasmid was performed using Lipofectamine 2000 according to manufacturer's protocol. Twenty four hours after transfection, cells were collected and subjected to Western blotting.

### Chromatin immunoprecipitation-sequencing (ChIP-seq)

Ten million WT and KO BEAS-2B cells, A549 cells, and/or MDA-MB-231 cells were fixed and subjected to immunoprecipitation using ChIP-grade antibodies against H3K4me3, H3K9me3 H3K27me3, H3R8me2a, and/or H4K20me3. All antibodies were from Active Motif (Carlsbad, CA). ChIP and input/control DNA were prepared for sequencing using Illumina NextSeq 500, and the 75-nt sequence reads (tags) were aligned to the reference genome hg19 using the Burrows-Wheeler Aligner (BWA) algorithm with default settings. Reads were extended at their 3'-ends to a length of 200 base pair (bp) fragments in a size-selected library. The densities of fragments (signal map) were determined by the number of fragments in each 32-nt bins along genome. The tag number of samples within a comparison group was normalized by random sampling to the number of tags in the smallest sample. Data was visualized by the University of California, Santa Cruz (UCSC) genome browser.

### RNA sequencing (RNA-seq)

Two million WT or KO BEAS-2B cells, A549 cells and/or MDA-MB-231 cells were collected according to Active Motif's protocol. Total RNA was isolated, and the RNA quality/integrity was assessed using an Agilent Bioanalyzer. Preparation of libraries and RNA sequencing were conducted by Active Motif Inc. based on Illumina platform. Reads were mapped to the reference genome hg19. Cufflinks methods were used for transcripts assembly and differentially expression analysis. The expression value of each gene was normalized by geometric FPKMs (Fragments per Kilobase Million). Data was visualized by IGV genome browser. Vennerable R package was used to generate the Venn diagram.

### Human Chemokine Array

WT or KO BEAS-2B cells were seeded in 12-well plate (1.3 × 10^5^/well) and cultured in Dulbecco's modified Eagle's medium (DMEM) with 5% FBS. After 24h, media were aspirated, and cells were washed with PBS, followed by addition of 1 ml of serum-free DMEM. After 24h, cell culture media were collected and then centrifuged at 1,500 rpm for 10 min at 4°C. The levels of chemokines were determined by Human Chemokine Array (R&D systems, Minneapolis, MN) according to manufacturer's protocol.

### Statistics

The quality control of ChIP-seq and RNA-seq data were made by Active Motif (Carlsbad, CA). At least three independent experiments were performed for Western blotting and chemokine array. In some experiments, statistical significance of the results was determined using the unpaired Student's t-test.

## Results

### Knockout of mdig increases global levels of repressive histone trimethylation

**Figure 1:**
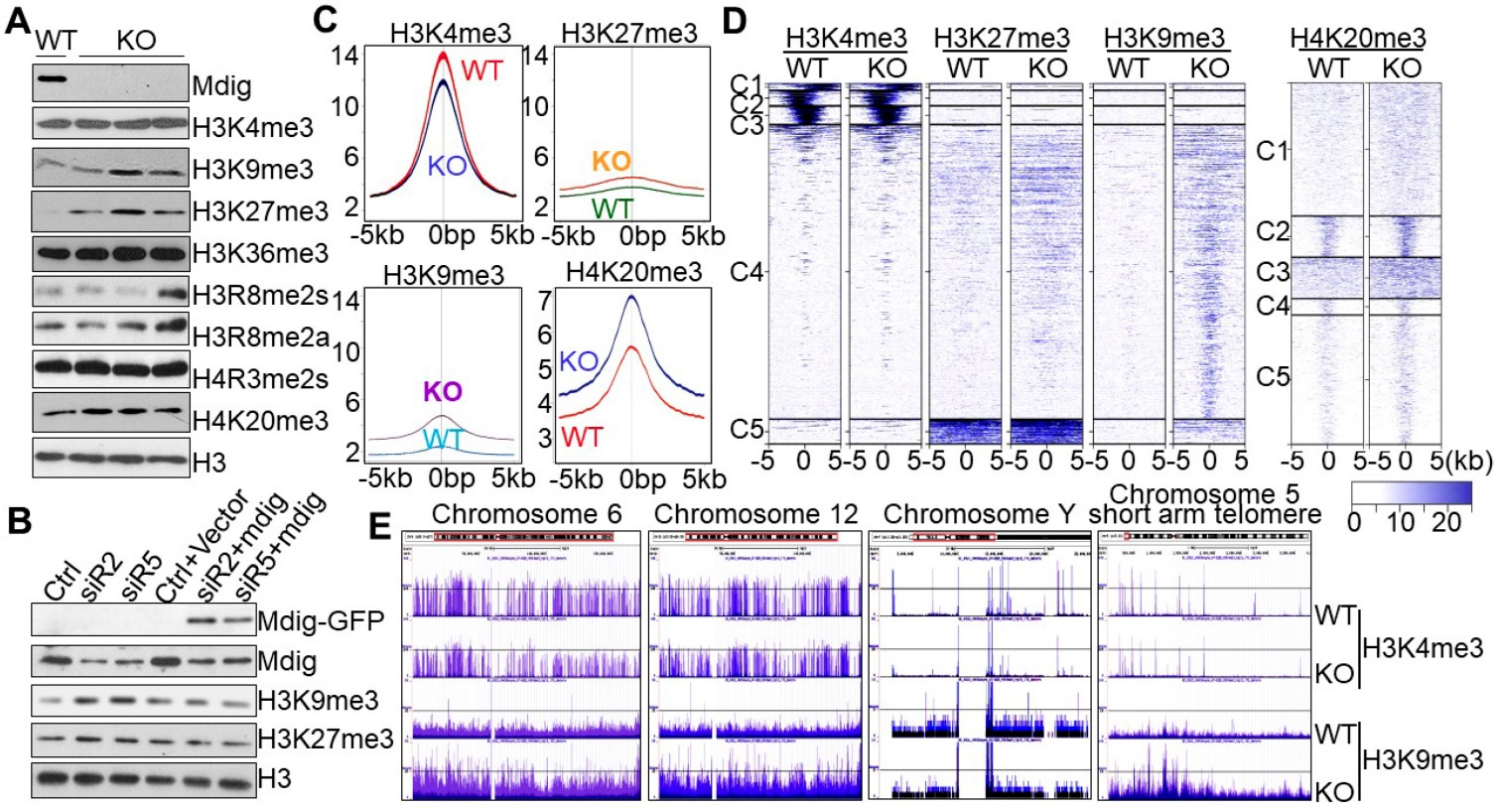
Knockout of mdig up-regulates repressive histone methylation markers. **A.** Examination of the methylation states of the indicated lysine markers in histone H3 of the BEAS-2B cells. WT: the cells subjected to CRISPR-cas9-based mdig targeting but failed in mdig knockout; KO: the cells with successful mdig knockout through CRISPR-cas9 approach. Data are representative of one of the two WT colonies and three of six KO colonies. **B.** Mdig silencing and rescue experiment. The BEAS-2B cells were transfected with the indicated mdig siRNAs alone or in combination with the GFP-tagged mdig expressing vector. **C.** Histone methylation profiles of ChIP-seq for H3K4me3, H3K27me3, H3K9me3, and H4K20me3 between WT and mdig KO cells. Average plots of the merged peak regions were shown. **D.** Heatmaps of the merged peak distribution of H3K4me3, H3K27me3, H3K9me3, and H4K20me3 between WT and mdig KO cells. **E.** All chromosomes showed an enrichment of H3K9me3 following mdig knockout (KO). Representative enrichment diagrams of H3K9me3 are shown for chromosomes 6, 12, Y, and the short arm telomere region of chromosome 5 between WT and KO cells

To knockout mdig, we transfected the human bronchial epithelial cell line BEAS-2B with pSpCas9-2A-Blast vector containing sgRNA that targets the third exon of the mdig gene and subsequently performed blasticidin selection for two weeks. Cell colonies were screened for mdig expression by both Westernblotting and RNA-seq, respectively (Figs. s1A and s1B). Profiling of histone methylation showed no appreciable changes in monomethylation or dimethylation of histone 3 lysine 4 (H3K4), H3K9, H3K27, or H3K36 in mdig knockout (KO) cells (Fig. s1C). However, a notable enhancement of the repressive histone trimethylation, including H3K9me3, H3K27me3, and H4K20me3, was observed in three individual KO cell colonies relative to the wild-type (WT) cells (Fig. s1C and Fig. 1A). There were no reproducible effects detected for mdig knockout on the levels of H3K4me3, H3K36me3, H3R8me2a/s, and H4R3me2s.

To additionally confirm the effects of mdig knockout on the repressive histone trimethylation, we also performed siRNA silencing and mdig rescue experiment in the mdig-silenced BEAS-2B cells, followed by immunoblotting to measure the levels of the repressive histone trimethylation markers, H3K9me3 and H3K27me3. As depicted in Fig. 1B, silencing mdig by two different siRNAs, siR2 and siR5, reduced the protein level of mdig, resulting in a notable enhancement of H3K9me3 and H3K27me3. This enhancement was prevented by re-expression of the exogenous mdig protein.

To investigate the methylation status of the chromatin-incorporated histone proteins, rather than the total histone proteins in the cells, we next performed ChIP-seq using WT and KO cells and antibodies against H3K4me3, H3K9me3, H3K27me3, H4K20me3, and H3R8me2a. All analyses for the enrichment distribution of these histone methylation markers suggested a substantial increase of H3K9me3, H3K27me3 and H4K20me3 in gene bodies, promoter regions and merged peaks in the KO cells relative to WT cells (Figs. 1C and 1D). In contrast, a significant reduction of H3K4me3, the active trimethylation marker of histone H3, was observed in the KO cells (Fig. 1C). No significant differences were detected in the level of H3R8me2a between WT and KO cells (Fig. s2). We speculate that the reduction of H3K4me3 in chromatin in the KO cells might be a secondary response to the increased levels of H3K9me3, H3K27me3 and H4K20me3 that antagonize access of the H3K4 methyltransferases to the chromatin. The most enriched repressive histone methylation marker is H3K9me3 in the mdig-depleted KO BEAS-2B cells. In fact, visualization of the ChIP-seq data using UCSC Genome Browser revealed that except Y chromosome, all other chromosomes gained a pronounced increase in the level of H3K9me3 in the entire chromosome region of the KO cells, as exampled for chromosomes 6 and 12 in Fig. 1E. Further analysis suggested that the gene poor regions of the chromosomes exhibited the greatest enrichment of H3K9me3 following mdig knockout, as depicted for the short arm telomere of chromosome 5 (Fig. 1E).

### Reduced expression of cell proliferative and/or stemness genes in the mdig KO cells

**Figure 2:**
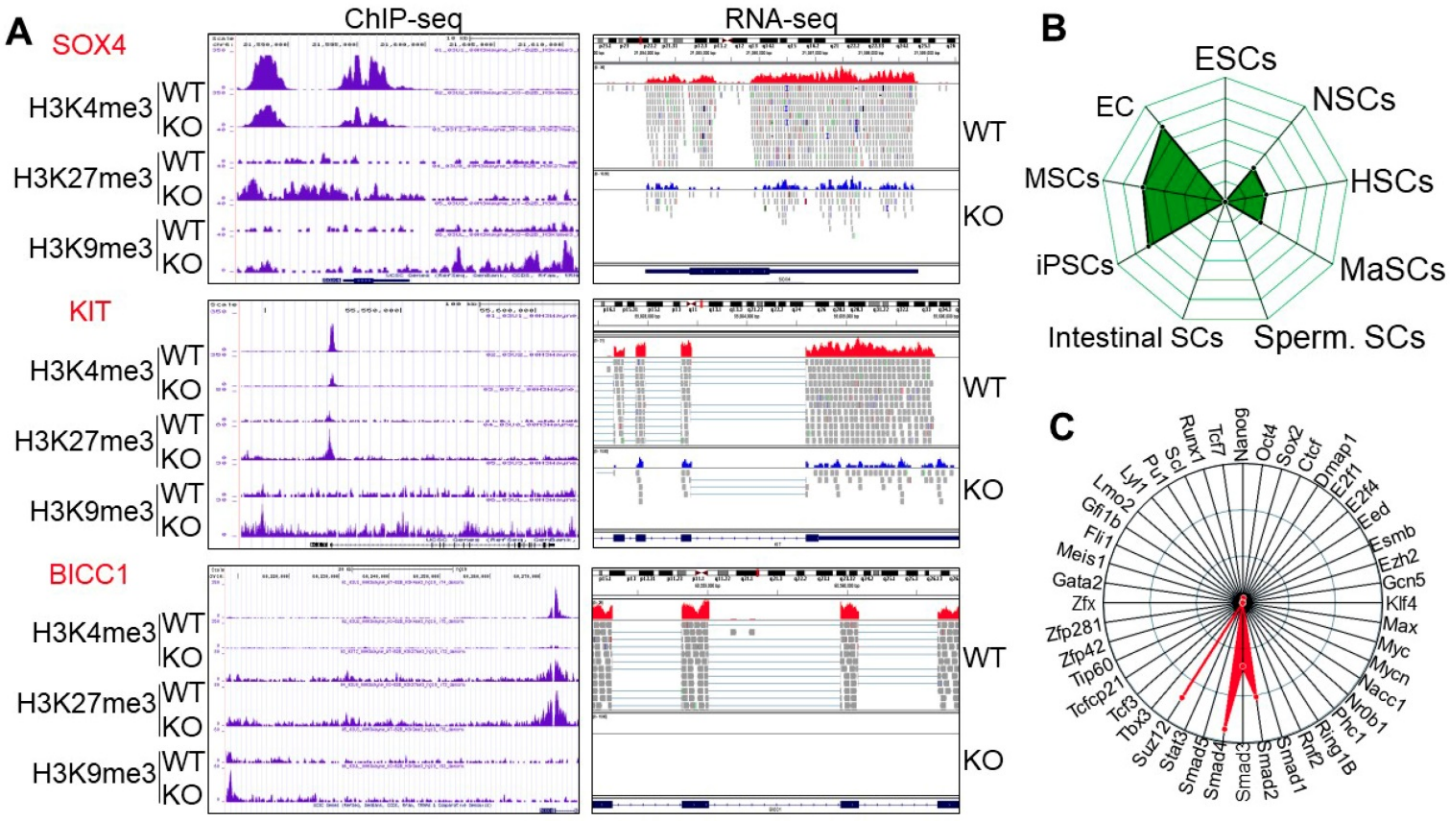
** Knockout of mdig enriches H3K9me3, H3K27me3, and/or reduces H3K4me3 on the genes in cell growth and stemness. A.** Representative histone methylation profiles and expression of the indicated stemness genes in WT and KO cells as determined by ChIP-seq and RNA-seq, respectively. **B.** Stem cell signature assay for the genes with an increased enrichment of H3K9me3 following mdig knockout as determined by StemChecker software. **C.** Transcription factor target analysis of the genes with an increased enrichment of H3K9me3 following mdig knockout as determined by StemChecker software.

To gain insight into which sets of genes are impacted by the altered histone methylation profiles due to knockout of mdig, we next performed gene pathway analysis for the biological functions (BP) of genes that showed significant increases of H3K9me3, H3K27me3, and/or loss of H3K4me3. Among the top 100 pathways revealed by g:Profiler program, development-related pathways are accounted for 43%, followed by 19% of morphogenesis, 12% of adhesion, and 8% of differentiation (Fig. s3A). The top 3 pathways are the genes that play critical roles in the development of anatomical structure, system and organism. Many genes in this group are involved in cell growth and self-renewal of the adult stem cells or cancer stem cells, including APCDD1, BICC1, CLEC11A, FGFR2, HLF, ISL, KIT, PDGFB, PDGFRA, PPARG, S1PR1, SAMD11, SOX4, SPOCK1, WNT5A, etc (Fig. 2A, Fig. s4 and Fig. s5). RNA-seq analysis showed a 2- to 20-fold reduction of these genes in KO cells as compared to the WT cells. ChIP-seq results for histone methylation revealed that most of these genes showed an increase of H3K9me3 and H3K27me3 in the gene body, promoter, and/or upstream of the gene loci, along with a notable depletion of H3K4me3 in the transcription start site (TSS) in the KO cells (Fig. 2A and Fig. s4). These data imply that mdig might be important for controlling the expression of genes in cell growth and stemness of normal or cancer stem cells. Deletion of mdig resulted in an enhanced enrichment of H3K9me3 and H3K27me3 in genes that showed decreased expression in KO cells. Additional bioinformatics analysis for mdig-regulated genes by StemChecker suggested that this group of genes is enriched in induced pluripotent stem cells (iPSC), mesenchymal stem cells (MSC), and embryonic cancers (EC), and to a lesser degree, in neural stem cells (NSC), hematopoietic stem cells (HSC), and mammary stem cells (MaSC), but not in embryonic stem cells (ESC), intestinal stem cells, or spermatogonial stem cells (Sperm. SC) (Fig. 2B). This result may be relevant to our previous finding showing reduced number of Th17 cells in the lung of mdig knockout mice in response to silica-induced pulmonary fibrosis 21, since Th17 cells retain some stem cell-like features 24. Transcription factor network analysis indicated that majority of these genes are transcriptionally regulated by Suz12, Smad4, Smad2 and Smad3 (Fig. 2C).

### Mdig regulates genes critical in lung fibrosis

**Figure 3:**
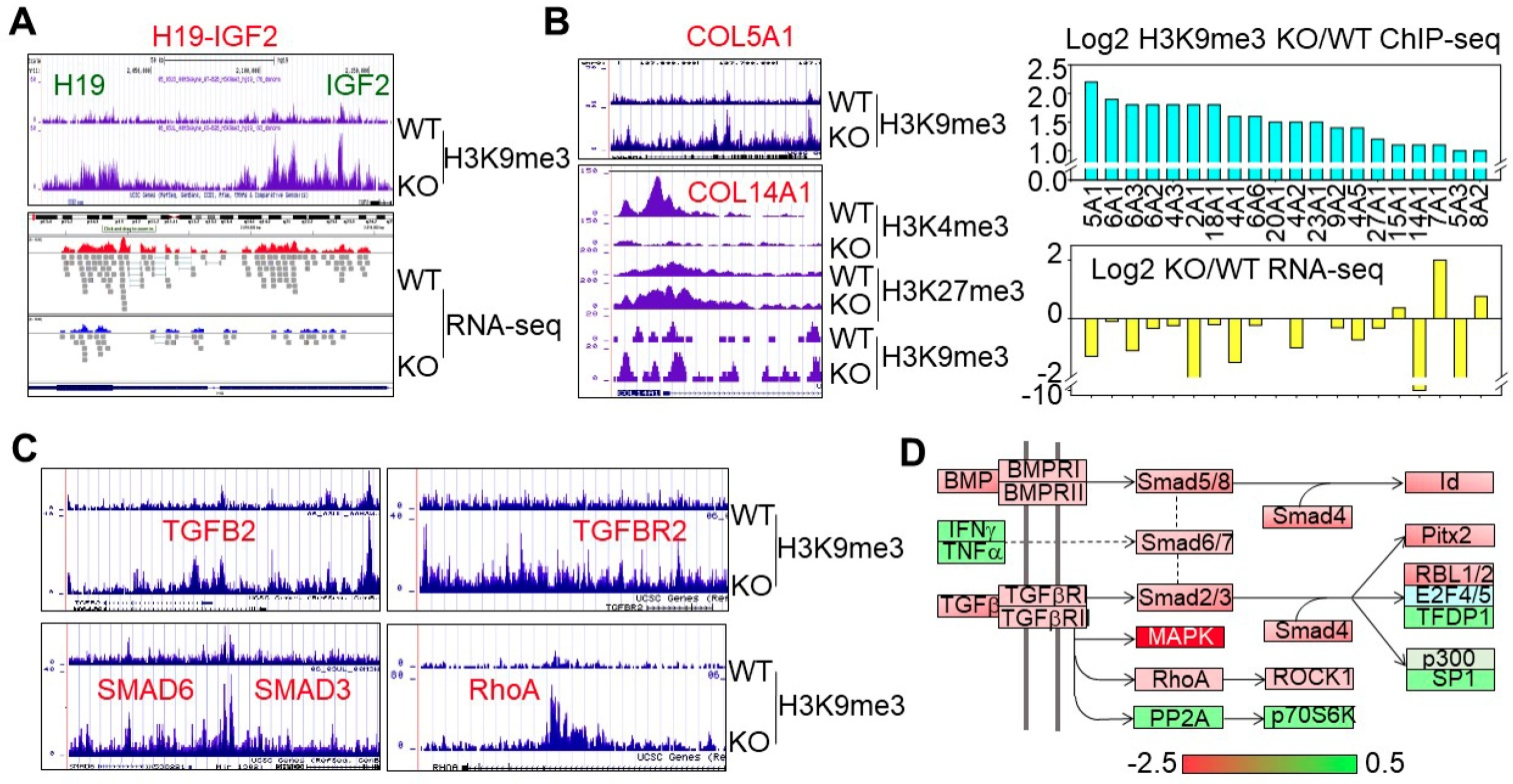
Disruption of mdig gene elevates the level of H3K9me3 on the imprinted H19-IGF2 loci and the genes involved in lung fibrosis. **A.** ChIP-seq shows an increased enrichment of H3K9me3 in the imprinting control region and promoter regions of the H19-IGF2 loci in the mdig KO cells. Bottom panel shows that this enrichment of H3K9me3 is correlated with a reduced expression of H19 as evidenced by RNA-seq. **B.** Mdig KO cells exhibited enhanced H3K9me3, H3K27me3, and/or reduced H3K4me3 on the collagen genes involved in lung fibrosis. This histone methylation profile is associated with an overall reduced expression of collagen genes in the KO cells as determined by RNA-seq. **C & D.** Mdig knockout increased H3K9me3 (**C**) and decreased expression (RNA-seq, **D**) of the key genes in TGFβ signaling.

Mdig was originally identified in alveolar macrophages from the coal miners who suffered from certain degrees of coal workers pneumoconiosis, lung fibrosis or other chronic lung diseases 1. We previously reported that targeted deletion of mdig in mice protected against silica-induced lung fibrosis 21. Molecular Function (MF) analysis of the H3K9me3-enriched genes in KO cells suggested that the top ranked genes are those involved in extracellular matrix structural constituent and binding proteins for growth factor, heparin, G-proteins, integrin, cytoskeletal proteins, etc (Fig. s3B). All of these genes are important in tissue remodeling and fibrosis. In ChIP-seq, we noted that knockout of mdig in human bronchial epithelial cells significantly elevated the levels of H3K9me3 and/or H3K27me3, and decreased the expression of the genes that contribute to lung fibrosis (Fig. 3). The long non-coding RNA H19 is a paternally imprinted gene and its overexpression has been linked to fibrotic diseases of the lung 25, liver 14, 26, and kidney 15. Our earlier studies suggested an increase or decrease of H3K9me3 in the imprint control region of the H19-IGF2 loci in mdig silenced cells or mdig overexpression cells, respectively 5. In mdig KO cells, a 3- to 5-fold increase of H3K9me3 was noted in the promoter and imprint control region of the H19-IGF2 gene, along with a considerable reduction of H19 expression as determined by RNA-seq (Fig. 3A). The most important histological feature of lung fibrosis is the accumulation of collagen proteins in the interstitium. Among 37 collagen genes detected in ChIP-seq, 20 of which showed a significant gain of H3K9me3 in mdig KO cells, and 16 out of these 20 genes were down-regulated as determined by RNA-seq in mdig KO cells (Fig. 3B), which was further confirmed by proteomic analysis suggesting a significant decrease of the collagen proteins in the KO cells (data not shown). A sub-set of fibrotic genes is the genes in the TGFβ signaling pathway. The gene loci of TGFB2, TGFBR2, SMAD6, SMAD3, and RhoA showed an increase of H3K9me3 in mdig KO cells (Fig. 3C), which was accompanied by a decreased expression as determined by RNA-seq (Fig. 3D).

### Knockout of mdig gene in mice ameliorates silica-induced lung fibrosis

**Figure 4:**
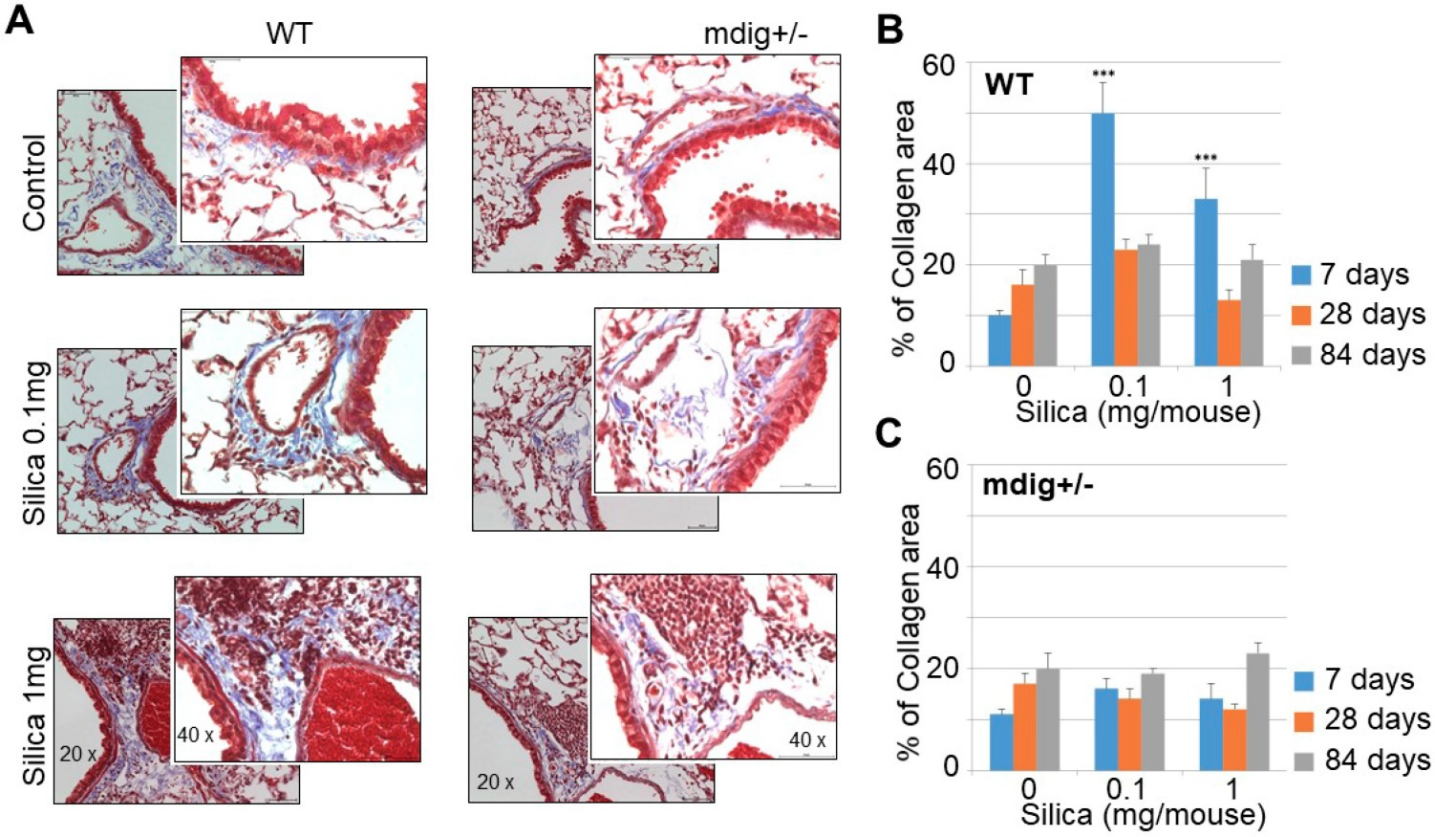
** Knockout of mdig gene in mice prevents silica-induced lung fibrosis. A.** Immunohistochemistry of collagen accumulation in the lung tissues from WT and mdig^+/-^ mice. Representative sections from 10 mice per treatment group per genotype at 7 days post exposure are shown. **B & C.** Semi-quantification of the levels of lung fibrosis induced by silica at the indicated days after silica treatment in WT (B) and mdig^+/-^ mice (C). Relative quantification of the collagen accumulation per 10 random microscopic fields (~0.1256 mm^2^ ) was shown. *P < 0.05 compared with respective genotypes (mdig^+/-^ vs WT).

Both ChIP-seq and RNA-seq clearly indicated regulation of mdig on the repressive histone trimethylation and expression of the genes in extracellular matrix formation and fibrosis. To support this notion further, we re-analyzed the immunohistochemistry data of the lung tissues collected from both the wild-type (WT) and heterozygous mdig knockout (mdig^+/-^) mice in control and silica treatment conditions, respectively. In a detailed time-course study by treating the mice with 0.1 and 1 mg/mouse of silica through pharyngeal aspiration, respectively, we found that both doses of silica induced a significant lung fibrosis as indicated by Masson's Trichome staining for the accumulated collagens in the lung interstitium in WT mice. The highest degree of lung fibrosis occurs after 7 days of silica treatment (Fig. 4). In the heterozygous mdig knockout (mdig+/-) mice, in contrast, neither dose of silica induces significant lung fibrosis after 7, 28 and 84 days of silica treatment, suggesting that knockout of mdig gene is protective for lung fibrosis.

### Opposite regulation of mdig on cell motility genes and chemokine genes

**Figure 5:**
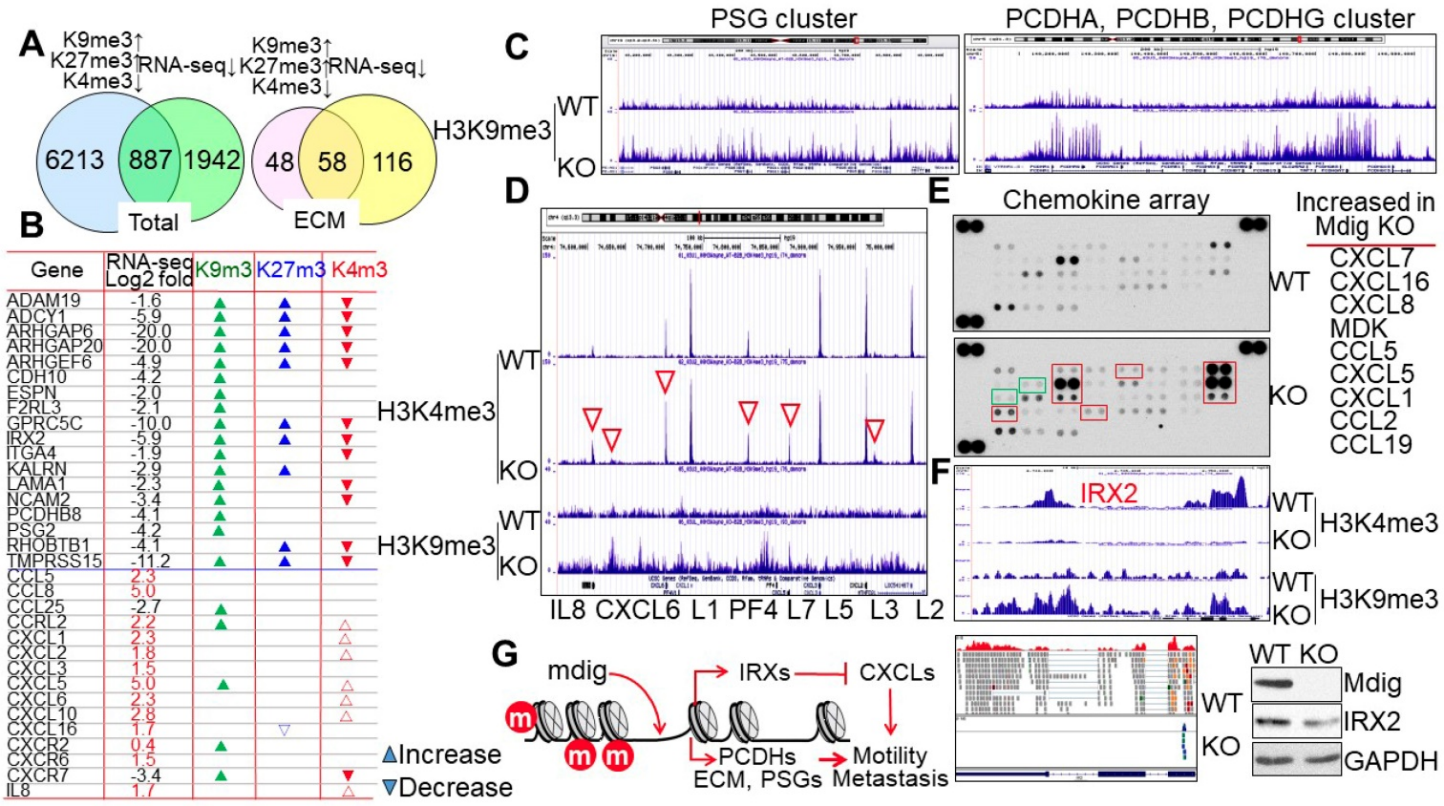
Mdig differentially regulates the expression of genes contributing to cell motility, migration, or metastasis. **A.** Venn diagrams show overlapping of total genes (left) and extracellular matrix (ECM) genes (right) between ChIP-seq showing gain of H3K9me3, H3K27me3, and/or loss of H3K4me3, and RNA-seq showing a decreased expression. **B.** List of some typical cell motility or chemotaxis genes. The expression of these genes in KO cells relative to WT cells as determined by RNA-seq was indicated by Log2 fold changes. Up and down triangles represented increase and decrease of the indicated histone methylation markers in the mdig KO cells, respectively. **C.** ChIP-seq spectrum shows gain of H3K9me3 of the pregnancy specific beta-1-glycoprotein (PSG) gene cluster on chromosome 19 (left) and protocadherin (PCDH) cluster genes on chromosome 5 (right) in mdig KO cells. **D.** Mdig knockout enhances both H3K4me3 (red triangles) and H3K9me3 in the chemokine gene cluster on chromosome 4. **E.** Chemokine array shows elevation of chemokine expression in the mdig KO cells relative to the WT cells. Those chemokines with a significantly increased expression in KO cells were marked by red boxes and listed at the right of the array panels. Green boxes depicted two marginally increased chemokines, CCL7 and CCL1, in the KO cells. Representative image of three independent arrays were shown. **F.** Loss of IRX2 gene expression in mdig KO cells due to increase in H3K9me3 and decrease in H3K4me3. The decreased expression of IRX2 was verified by both RNA-seq and Westernblotting. **G.** Possible mechanism of mdig regulation on cell motility, migration and metastasis: mdig down-regulates H3K9me3 and H3K27me3 (marked in red circles) on the chromatin, to facilitate expression of gene families of PCDH, ECM, PSG, and others in cell adhesion, G-protein signaling, or cytoskeleton organization, which may promote cell motility or metastasis. At the same time, demethylation of H3K9me3 and H3K27me3 by mdig induces expression of IRX family genes, which served as major transcription repressors for the chemokines, therefore, antagonizes cell migration or metastasis.

The methylation status of histone proteins is one of multiple regulatory mechanisms for chromatin accessibility and gene expression. It is not surprising, therefore, to note that while many genes exhibited increased levels of H3K9me3, H3K27, and/or loss of H3K4me3 in the KO cells, RNA-seq did not reveal down-regulation of all of these genes. A comparison of genes with gain of H3K9me3/H3K27me3 and/or loss of H3K4me3 with the repressed genes detected in RNA-seq suggested that only about 12% of these gene in ChIP-seq are overlapped with the genes in RNA-seq (Fig. 5A, left). However, when we compared the genes involved in extracellular matrix (ECM) formation, cell motility and G-protein signaling in both ChIP-seq and RNA-seq, we discovered that about 55% of genes in ChIP-seq are overlapped with the genes in RNA-seq (Fig. 5A, right). The methylation profiles and expression levels of some of the typical genes in this group are listed in Fig. 5B. Some of these genes had also been implicated in the maintenance of the stem cells, e.g., the G protein-coupled receptor protein GPRC5C in neuroblastoma stem cells 27, the membrane-anchored disintegrin and metalloprotease domain protein ADAM19 in glioblastoma stem cells 28, the Rac/Cdc42 signaling protein ARHGEF6 in cancer stem cells 29, and type II cadherin protein CDH10 in germline stem cells 30. It is interesting to note that H3K9me3 was greatly enriched in several gene clusters for cell surface glycoproteins and protocadherins (PCDH) in the KO cells, such as the pregnancy specific beta-glycoprotein (PSG) cluster containing PSG3, PSG8, PSG1, PSG6, PSG7, PSG11, PSG2, PSG5, PSG4, PSG9, and CD177 on chromosome 19, and the PCDH cluster containing PCDHAs, PCDHBs, and PCDHGs on chromosome 5 (Fig. 5C). This finding suggests that loss of mdig reduces the expression of these cell adhesion or motility genes due to the enrichment of H3K9me3 that promotes formation of heterochromatin. However, another cluster of genes encoding chemokines for cell migration and cancer cell metastasis, not only showed moderate enrichment of H3K9me3, but also a pronounced gain of H3K4me3 in the KO cells, including IL8, CXCL6, CXCL1, PF4, CXCL5, CXCL3, and CXCL2 on chromosome 4 (Figs. 5B and 5D). Both chemokine arrays and RNA-seq demonstrated a remarkable increase of these chemokines in KO cells relative to WT cells (Fig. 5E and Fig. s6), indicating that mdig is an inhibitory factor for the expression of some key chemokines. The iroquois homeobox 2 (IRX2) had been demonstrated as a transcription repressor for the chemokines 31. The increased expression of chemokines in the KO cells might be an indirect effect of mdig knockout, due to the loss of IRX2. Indeed, ChIP-seq results indicate that the IRX2 gene locus not only showed a notable gain of H3K9me3, but also a complete loss of H3K4me3 in the KO cells, which was correlated with a diminished expression of IRX2 as determined by both RNA-seq and immunoblotting (Fig. 5F and Fig. s7). The net effect of mdig on cell motility or metastasis, thus, may be dependent on the balance between cell surface adhesion molecules and the chemokines (Fig. 5G). The upregulation of chemokines in mdig KO cells may provide complementary evidence supporting our previous observations showing that mdig silencing by siRNAs enhanced cancer cell migration and invasion 17, and the lower level of mdig expression in the metastatic breast cancers 20.

### Knockout of mdig increases repressive trimethylation markers and induces differentiation of the cancer stem cells

**Figure 6:**
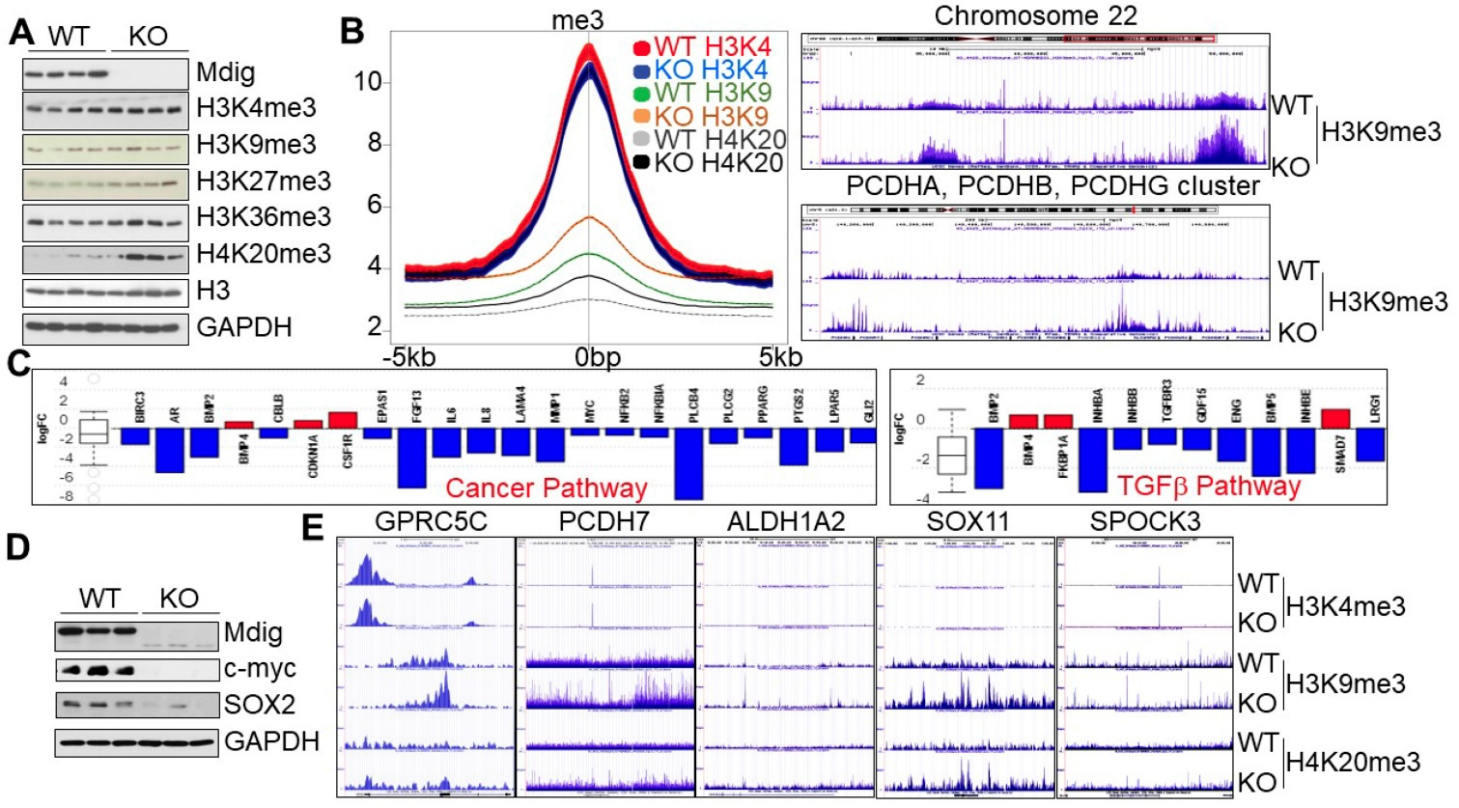
** Mdig knockout enriches repressive histone methylation markers and induces differentiation of the cancer stem cells (CSCs). A.** Westernblotting for mdig protein and the indicated histone methylation markers in WT and mdig KO MDA-MB-231 cells. **B.** Histone methylation profiles of ChIP-seq for H3K4me3, H3K9me3 and H4K20me3 between WT and mdig KO cells. Average plots of the merged peak regions were shown. **C.** Pathway analysis of the RNA-seq data shows diminished expression of the genes in cancer pathway (left) and the TGFβ signaling (right) in the mdig KO MDA-MB-231 cells. **D.** Depletion of mdig reduced expression of c-myc and SOX2 in MDA-MB-231 cells. **E.** Gain of H3K9me3 and/or H4K20me3 on the indicated stemness genes for CSCs in the mdig KO MDA-MB-231 cells.

To investigate whether knockout of mdig exhibits similar effects on histone methylation in human cancer cells, in addition to the non-cancerous cells in above experiments, we also created mdig KO cells by CRISPR-Cas9 gene editing from MDA-MB-231 (Fig. 5) and human lung cancer cell line, A549, respectively. MDA-MB-231 cells are triple negative breast cancer cells with some characteristics of the cancer stem cells (CSCs) 32. Among the four WT and four mdig KO MDA-MB-231 cell clones we screened, the KO clones showed upregulation of H3K9me3, H3K27me3, and H4K20me3, without appreciable changes in H3K4me3 and H3K36me3 (Fig. 6A). Data from ChIP-seq suggested a marginal reduction of H3K4me3 in the KO cells. In contrast, a significant gain of H3K9me3 and H4K20me3 was observed in the KO cells relative to the WT cells (Fig. 6B). The increased enrichment of H3K9me3 can be detected on all of the autosomes and X chromosome, as exampled for chromosome 22 and the PCDH gene clusters (Fig. 6C, right panel). Gene expression profiling as determined by RNA-seq unraveled a significantly reduced expression of the genes in cancer pathway (Fig. 6C, left panel) and TGFβ signaling (Fig. 6C, right panel). Most importantly, the KO cells showed a significant decrease of c-myc and Sox2, two key stemness factors for stem cells and CSCs (Fig. 6D). Furthermore, substantial enrichment of H3K9me3 and/or H4K20me3 on several key genes critical for the CSCs, including GPRC5C, PCDH7, ALDH1A2, SOX11, and SPOCK3 (Fig. 6E). These data, thus, clearly indicate that knockout of mdig induces differentiation of the CSCs through increasing the repressive histone methylation and minimizing expression of the stemness genes.

### Enhanced H3K27me3 in mdig KO A549 cells

**Figure 7:**
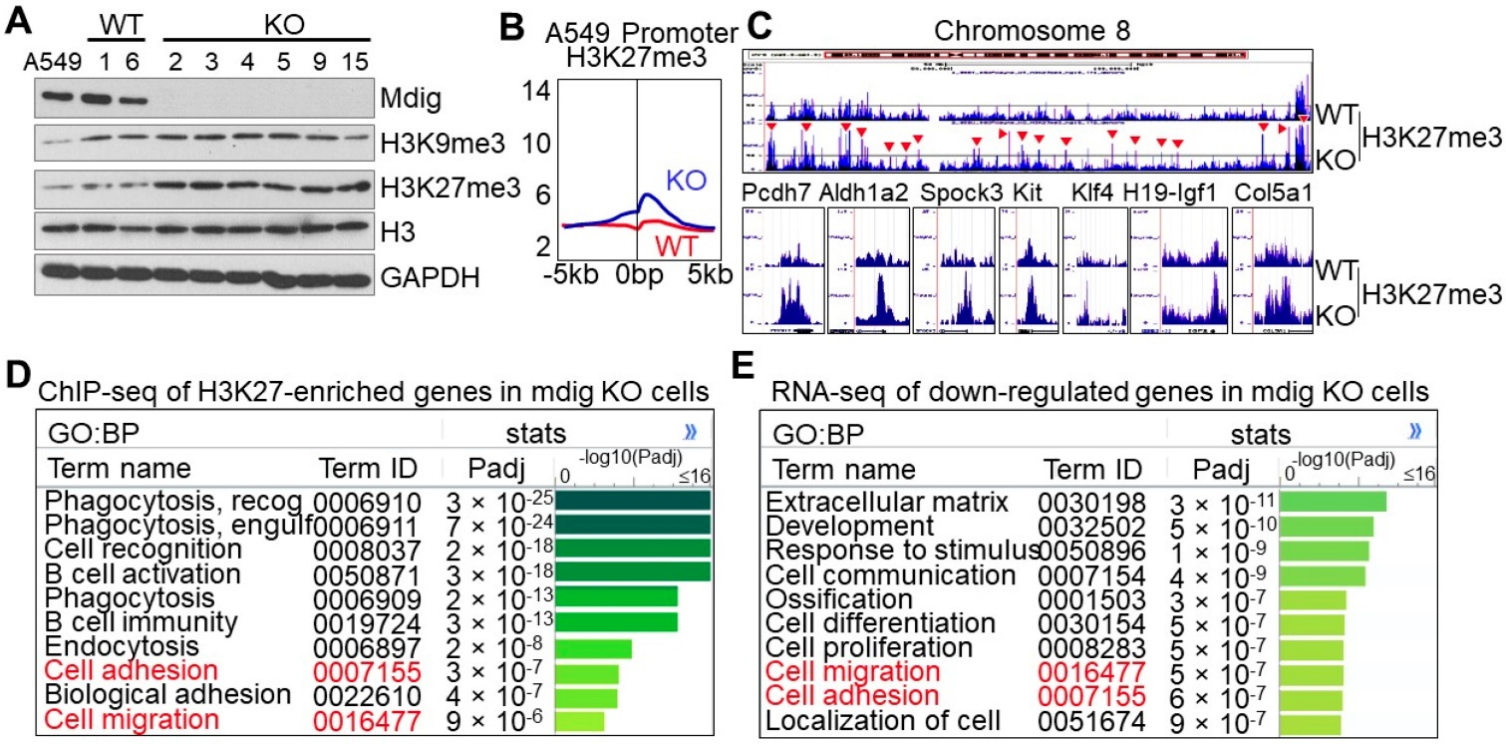
** Knockout of mdig enhances H3K27me3 in A549 cells. A.** The protein levels of mdig, H3K9me3, H3K27me3, H3, and GAPDH between WT and mdig KO cells were determined by Westernblotting. **B.** Global level of H3K27me3 in WT and mdig KO cells were determined by ChIP-seq. Data show enrichment of H3K27me3 in the promoter region of genes. **C.** Gain of H3K27me3 on the entire chromosome and the indicated genes in the mdig KO cells. **D.** Pathway analysis of the H3K27me3-enriched genes in mdig KO cells. **E.** Pathway analysis of the down-regulated genes in mdig KO cells as determined by RNA-seq.

Owing to the unique characteristics of gene expression in any given type of cells, the histone methylation profiles may be different following mdig knockout in different cells. This is true for the cells of lung cancer cell line A549. Knockout of mdig by CRISPR-Cas9 gene editing promoted a significant enrichment of H3K27me3, but not other repressive histone trimentylation markers (Fig. 7A and data not shown). In agreement with immunoblotting, we found a pronounced enrichment of H3K27me3, but not others, in the promoter region of the mdig KO A549 cells (Fig. 7B). In fact, gain of H3K27me3 in the KO cells can be visualized at the entire chromosome level as exampled for chromosome 8 (Fig. 7C, upper panel). At the individual gene levels, again, we noted that mdig KO in A549 cells also enriched the repressive histone trimethylation marker, H3K27me3, on the genes important for cancer stem cells and fibrosis, including PCDH7, ALDH1A2, SPOCK3, KIT, KLF4, H19-IGF1, COL5A1, etc (Fig. 7C, bottom panels). Pathway analysis for the biological process (BP) of the H3K27me3-enriched genes in the KO A549 cells indicated that the top-ranked genes are in the cell phagocytosis, adhesion and migration pathways (Fig. 7D). The same analysis was applied to the down-regulated genes as determined by RNA-seq suggested that most of these genes are in the formation of extracellular matrix, development, cell migration, and cell adhesion (Fig. 7E). Thus, there is a significant correlation between ChIP-seq for H3K27me3 and RNA-seq, esp. for those genes important in cell adhesion and migration.

## Discussion

Genetic disruption of mdig gene is found to increase global levels of H3K9me3, H3K27me3 and H4K20me3, the three major repressive histone methylation markers, suggesting functional property of mdig in the demethylation processes of the histone proteins (Fig. 1). Importantly, many genes that are involved in cell growth, fibrosis and cell motility showed an overall enrichment of these repressive histone methylation markers, esp., the H3K9me3, following mdig depletion, indicating that mdig is one of the key genes contributing to inflammation, oncogenesis and cancer cell metastasis (Figs. 2, 3 & 4). The influence of mdig on the repressive histone methylation markers was additionally confirmed in both breast cancer cell line (Fig. 6) and lung cancer cell line A549 (Fig. 7). There is no appreciable effect of mdig detected on other histone methylation markers, including H3K36me3, H3R8me2a/s, H4R3me2s, dimathylations or monomethylations of H3K4, H3K9, H3K27, and H3K36. Intriguingly, a substantial reduction of H3K4me3 was noted in ChIP-seq of the mdig KO BEAS-2B cells and MDA-MB-231 cells, despite the level of total H3K4me3 protein was not changed as determined by Western blotting (Figs. 1A and 6A). Taken together, these data clearly suggest that mdig possesses demethylase-like activity toward the repressive histone methylation markers, including H3K9me3, H3K27me3 and H4K20me3.

Emerging evidence indicated pro-oncogenic roles of mdig in a number of human cancers, such as increased expression of mdig mRNA and protein in cancers and association of mdig expression levels with the prognosis and tumor recurrence of the cancer patients 2, 17, 18, 33, 34. The mechanisms of how mdig plays its roles in the development and pathogenesis of cancers remain to be fully understood. Several earlier studies demonstrated that mdig is able to promote cell proliferation and cell cycle transition 1, 3. Additional evidence further suggested an anti-apoptotic role of mdig in some types of cancer cells or tissues 6. In human glioblastoma, silencing mdig not only sensitized cell apoptosis induced by chemodrugs, but also caused DNA replication stress due to enrichment of H3K9me3 on the promoter regions of several DNA replication factors 6. In the mdig KO cells, some of these genes encoding pre-replication complex proteins and origin recognition complex proteins, indeed, exhibited either loss of H3K4me3, or significant gain of H3K9me3 (data not shown). Intriguingly, our previous study also showed physical interaction between mdig and the pre-replication complex proteins MCM5, MCM7 and the origin recognition complex protein ORC5 35.

One of the most important findings in our study is that mdig appears to be highly capable of regulating the expression of genes important for oncogenesis and/or the stemness of the cancer stem cells (CSCs), including SOX4, KIT, BICC1, PCDH7, Wnt5A, etc., through changing the histone methylation status of these genes (Figs. 2 and 6, and Fig. s4). Whereas the contribution of SOX4, KIT and Wnt family members to the generation or maintenance of CSCs had been well established, the importance of BICC1, CRISPLD1, PCDH7, SPOCK1/3, and others in CSCs is recognized just recently. BICC1, an RNA binding protein, and CRISPLD1, a cysteine-rich secretory protein, had been shown to be critical for maintaining the populations of pancreatic progenitor cells and hematopoietic stem cells, respectively 36, 37. PCDH7, an integral membrane protein of the cadherin superfamily, had been demonstrated to be essential for brain metastasis of the triple-negative breast cancer cells that acquired some features of CSCs 38. In both lung epithelial cells and MDA-MB-231 cells, the triple-negative breast cancer cell line, mdig knockout either enriched the repressive histone methylation markers or diminished H3K4me3 on the PCDH7 gene, along with a significant inhibition of PCDH7 gene expression as determined by either RNA-seq (Fig. s4) or Western blotting (data not shown). It is interesting to note that SPOCK1, another proteoglycan for cell-to-cell interaction, has also been shown to be highly capable of meditating brain metastasis of the lung cancer CSCs 39.

The mdig gene was originally identified from the people who were working in the mining industry and suffered from some chronic lung diseases associated with occupational exposure to mining dusts, including lung inflammation and fibrosis 1. In animal model, heterozygous knockout of mdig ameliorated lung fibrosis induced by silica particles 21. The findings that mdig knockout by CRISPR-Cas9 gene editing enriches H3K9me3 in the epithelial cells on genes of H19, collagens and the major components in TGFβ signaling provided the first direct evidence linking mdig to lung fibrosis. It is especially interesting to note the regulatory role of mdig on the members of the TGFβ signaling, as evidenced in both the lung epithelial cell-originated BEAS-2B cells (Figs. 3C & 3D) and the breast cancer cell line MDA-MB-231 cells (Fig. 6C). The importance of TGFβ in the cell lineage development of the Th17 cells had been well-established. As the key inflammatory T cells, Th17 cells produce pro-inflammatory cytokines involved in the pathogenesis of a spectrum of chronic inflammatory diseases and cancer 40. The findings that mdig regulates TGFβ signaling perhaps explain the observed phenomena that mice with mdig knockout exhibited a reduced number of Th17 cells infiltrated into the lung intestitium in response to silica instillation and the mdig promotes the expression of Th17 factors, including ROR-γt, Batf, and Irf4 21, 40. Furthermore, this finding also unraveled a forward feedback route between mdig and TGFβ. A recent comprehensive genetic study in BALB/C and C57BL/6 mice suggested that a single nucleotide polymorphism in the intron 1 region of mdig gene favors Smad3 binding, TGFβ responsiveness and mdig transcription 41.

The demethylation of mdig on H3K9me3 and H3K27me3 can be explained partially by the fact that mdig is capable of interacting with CBX3/CBX5 (HP1γ/HP1α) and RBBP4/RBBP7, respectively, in BEAS-2B cells as we reported previously 35. Both CBX3 and CBX5 can recognize and bind to H3K9me3 42. Accordingly, through physical interaction, CBX3 or CBX5 can bring mdig to the sites of H3K9me3. RBBP4 and RBBP7 are histone binding proteins and components of the PRC2 complex that promotes formation of H3K27me3 43. It is very likely, thus, that interaction of mdig with RBBP4 or RBBP7 may impair either the assembly or methyltransferase activity of the PRC2 complex. It is unknown at this moment on how mdig can also act on H4K20me3. The uncertainty of previous studies on the role of mdig in histone demethylation may be largely due to incomplete silencing of mdig by siRNAs/shRNAs or the artifacts of overexpression of mdig genes. Using a CRISPR-Cas9 approach, we achieved complete depletion of mdig protein in the KO cells. ChIP-seq data in combination with RNA-seq unequivocally demonstrated enhanced enrichment of H3K9me3, H3K27me3 and H4K20me3 in mdig KO cells, esp. for the genes in cell growth, stemness of the stem cells or cancer stem cells, lung fibrosis, and those involved in cell motility or cancer cell metastasis. Accumulating evidence indicated critical contributions of abnormal histone methylation and demethylation to a number of human malignancies 44, 45. Thus, understanding the intricate activity of mdig on histone methylation and gene expression, therefore, will help in designing new strategies for the targeted therapies of cancers.

## Supplementary Material

Supplementary figures and tables.
