# Supplementary material for: Mdig promotes oncogenic gene expression through antagonizing repressive histone methylation markers
Source: Theranostics. 2020 Jan 1;10(2):602–14. doi: 10.7150/thno.36220 (PMC6929976; doi:10.7150/thno.36220)
Supplement: Supplementary file 1 — Supplementary figures and tables. [file thnov10p0602s1.pdf]

sFig. 1

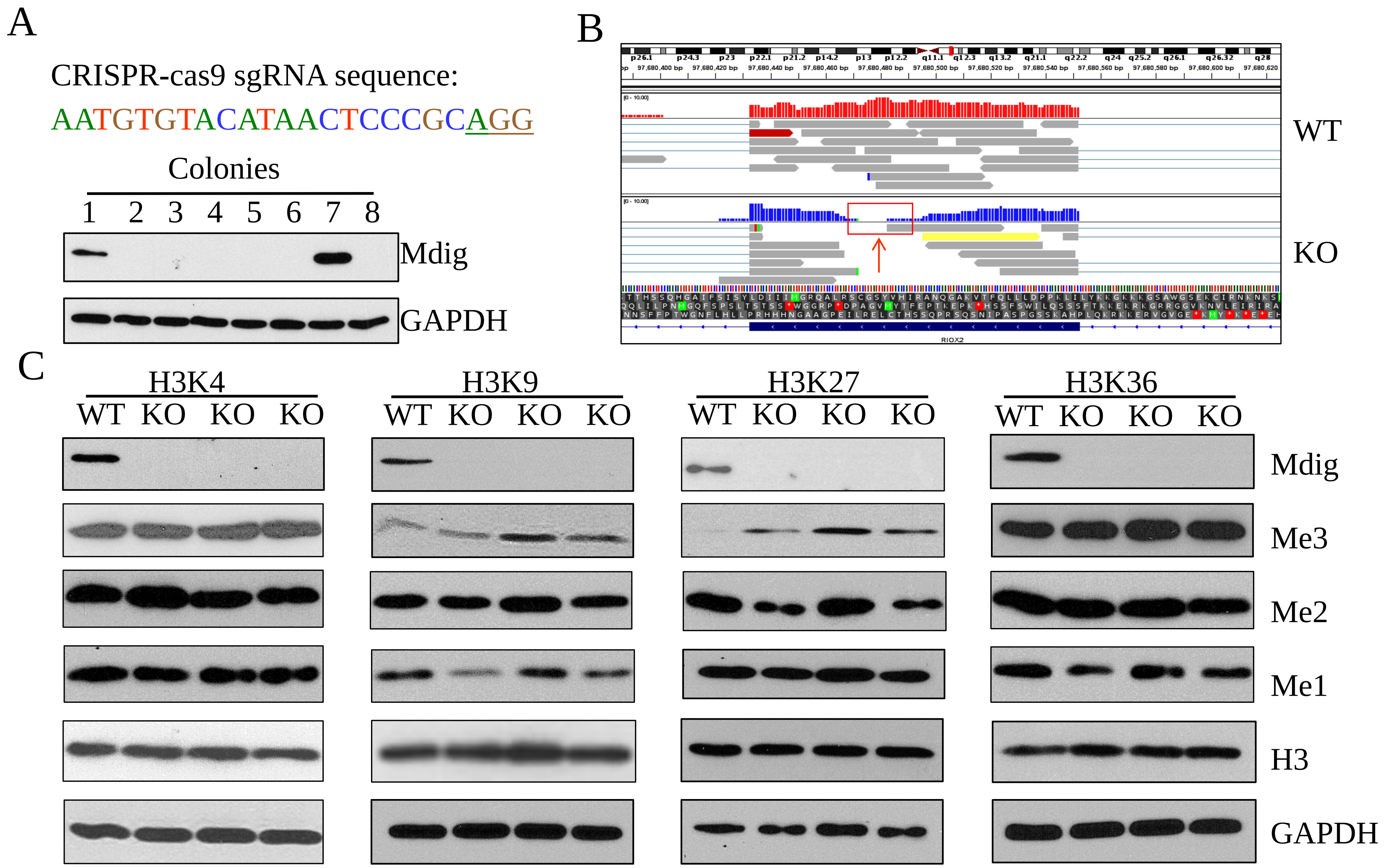
A
CRISPR-cas9 sgRNA sequence:
AATGTGTACATAACTCCCGCAGG
Colonies
1 2 3 4 5 6 7 8
Mdig
GAPDH
B
WT
KO
RIOX2
C
H3K4
H3K9
H3K27
H3K36
WT KO KO KO
Mdig
Me3
Me2
Me1
H3
GAPDH

sFig. 2

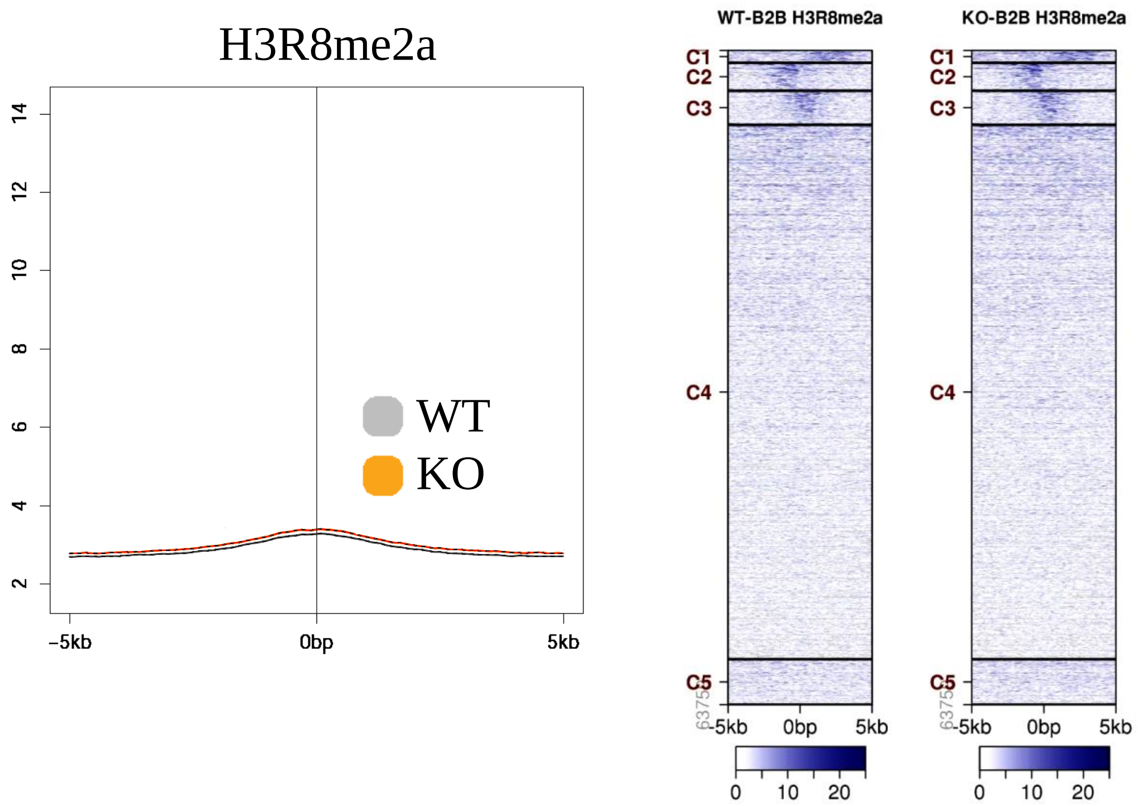
H3R8me2a
14
12
10
8
6
4
2
WT
KO
−5kb
0bp
5kb
WT-B2B H3R8me2a
KO-B2B H3R8me2a
C1
C2
C3
C4
C5
-5kb
0bp
5kb
0
10
20

sFig. 3

A

GO:BP		stats	
Term name	Term ID	p_{adj}	$-\log_{10}$(p... (0 – ≤16)
anatomical structure development	GO:0048856	6.770×10^{-21}	
system development	GO:0048731	2.161×10^{-20}	
multicellular organism development	GO:0007275	2.209×10^{-20}	
biological adhesion	GO:0022610	6.297×10^{-20}	
cell adhesion	GO:0007155	9.224×10^{-20}	
multicellular organismal process	GO:0032501	6.875×10^{-19}	
anatomical structure morphogenesis	GO:0009653	1.672×10^{-18}	
developmental process	GO:0032502	4.781×10^{-18}	
animal organ morphogenesis	GO:0009887	8.163×10^{-15}	
tissue development	GO:0009888	3.077×10^{-14}	
kidney development	GO:0001822	1.227×10^{-13}	
urogenital system development	GO:0001655	1.859×10^{-13}	
animal organ development	GO:0048513	2.507×10^{-13}	
renal system development	GO:0072001	4.230×10^{-13}	
regulation of multicellular organismal process	GO:0051239	1.292×10^{-12}	
circulatory system development	GO:0072359	1.683×10^{-12}	
nervous system development	GO:0007399	6.063×10^{-12}	
tube development	GO:0035295	1.499×10^{-11}	
cellular developmental process	GO:0048869	1.906×10^{-10}	
cell differentiation	GO:0030154	2.356×10^{-10}	
heart development	GO:0007507	6.159×10^{-10}	
tube morphogenesis	GO:0035239	8.210×10^{-10}	
tissue morphogenesis	GO:0048729	9.805×10^{-10}	
cellular component morphogenesis	GO:0032989	9.944×10^{-10}	
homophilic cell adhesion via plasma membran	GO:0007156	1.510×10^{-9}	
extracellular matrix organization	GO:0030198	2.080×10^{-9}	

B

GO:MF		stats	
Term name	Term ID	p_{adj}	$-\log_{10}$(p... (0 – ≤16)
extracellular matrix structural constituent	GO:0005201	5.591×10^{-10}	
glycosaminoglycan binding	GO:0005539	1.049×10^{-6}	
growth factor binding	GO:0019838	1.482×10^{-6}	
calcium ion binding	GO:0005509	2.172×10^{-6}	
heparin binding	GO:0008201	1.260×10^{-5}	
retinoic acid binding	GO:0001972	1.670×10^{-5}	
signaling receptor binding	GO:0005102	9.427×10^{-5}	
glucuronosyltransferase activity	GO:0015020	1.146×10^{-4}	
G-protein beta-subunit binding	GO:0031681	1.446×10^{-4}	
sulfur compound binding	GO:1901681	1.706×10^{-4}	
cytoskeletal protein binding	GO:0008092	3.792×10^{-4}	
integrin binding	GO:0005178	1.546×10^{-3}	
retinoid binding	GO:0005501	1.938×10^{-3}	
proteoglycan binding	GO:0043394	2.641×10^{-3}	
isoprenoid binding	GO:0019840	2.641×10^{-3}	
channel activity	GO:0015267	8.425×10^{-3}	
passive transmembrane transporter activity	GO:0022803	8.938×10^{-3}	
gated channel activity	GO:0022836	1.143×10^{-2}	
voltage-gated channel activity	GO:0022832	1.147×10^{-2}	
voltage-gated ion channel activity	GO:0005244	1.147×10^{-2}	
insulin-like growth factor binding	GO:0005520	1.224×10^{-2}	
collagen binding	GO:0005518	1.809×10^{-2}	
actin binding	GO:0003779	2.027×10^{-2}	
voltage-gated cation channel activity	GO:0022843	2.689×10^{-2}	
prostaglandin E receptor activity	GO:0004957	3.175×10^{-2}	
thrombin-activated receptor activity	GO:0015057	3.175×10^{-2}	

sFig. 4

Genes	KO vs WT
AURKC [1]	-2.6
BICC1 [2]	-7.1
CLEC11A [3]	-10
CRISPLD1 [4]	-20
FGFR2 [5]	-2.4
FLRT3 [6]	-20
HLF [7]	-2.1
HOXA7 [8]	-10
ISL1 [9]	-2
KIT [10]	-4
MSC [11]	-20
PCDH7 [12]	-20
PDGFB [13]	-3
PDGFRA [14]	-2.2
RIPPLY2 [15]	-10
S1PR1 [16]	-3.8
SAMD11 [17,18]	-2.3
SOX4 [19]	-3.6
SPOCK1 [20]	-7.8
SPOCK3 [21]	-3.5
WNT5A [22]	-20
WNT7B [23]	-2.6
WNT9A [24]	-2.2

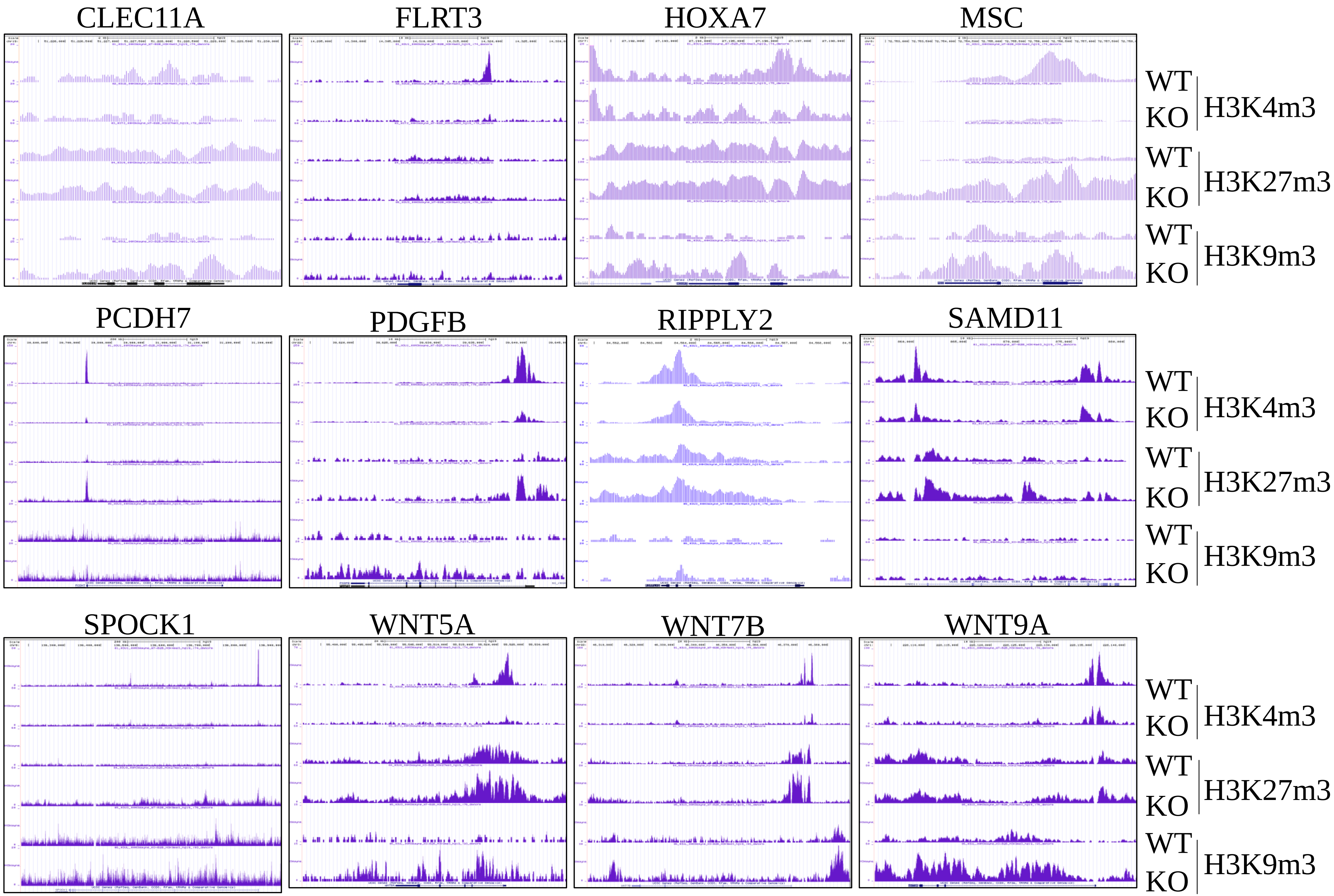

sFig. 5

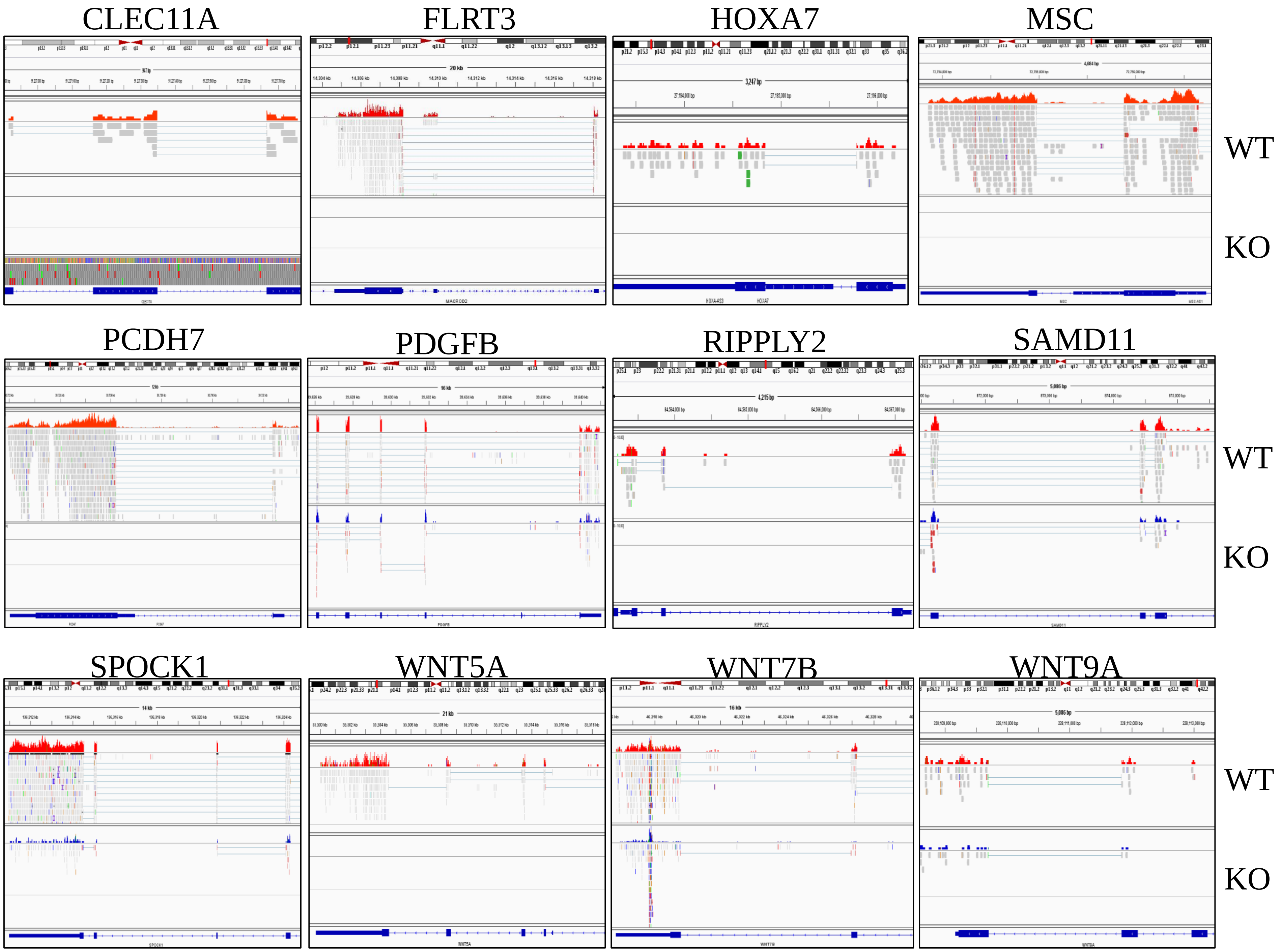
CLEC11A
FLRT3
HOXA7
MSC
PCDH7
PDGFB
RIPPLY2
SAMD11
SPOCK1
WNT5A
WNT7B
WNT9A
WT
KO
WT
KO
WT
KO

sFig. 6

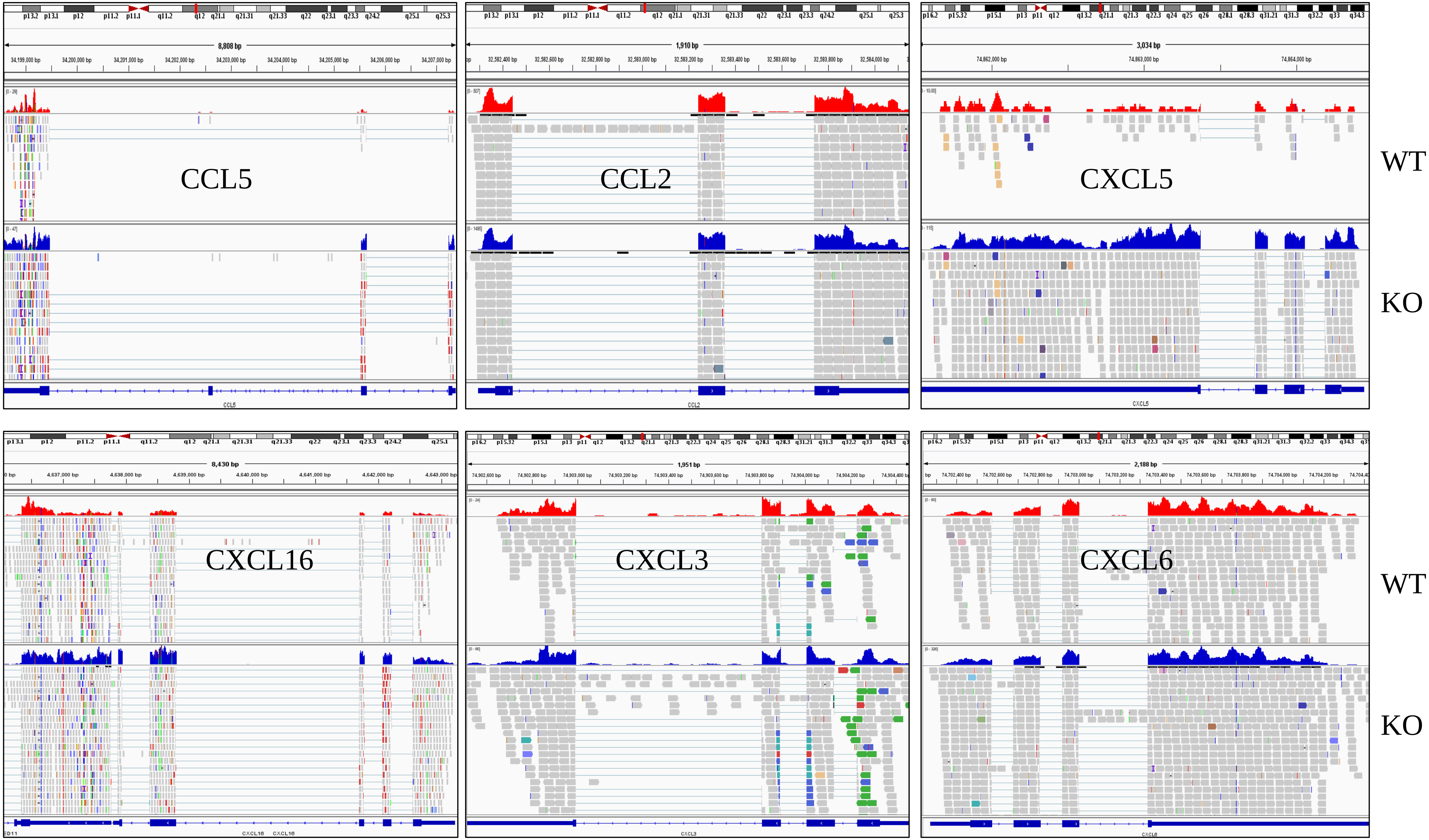

sFig. 7

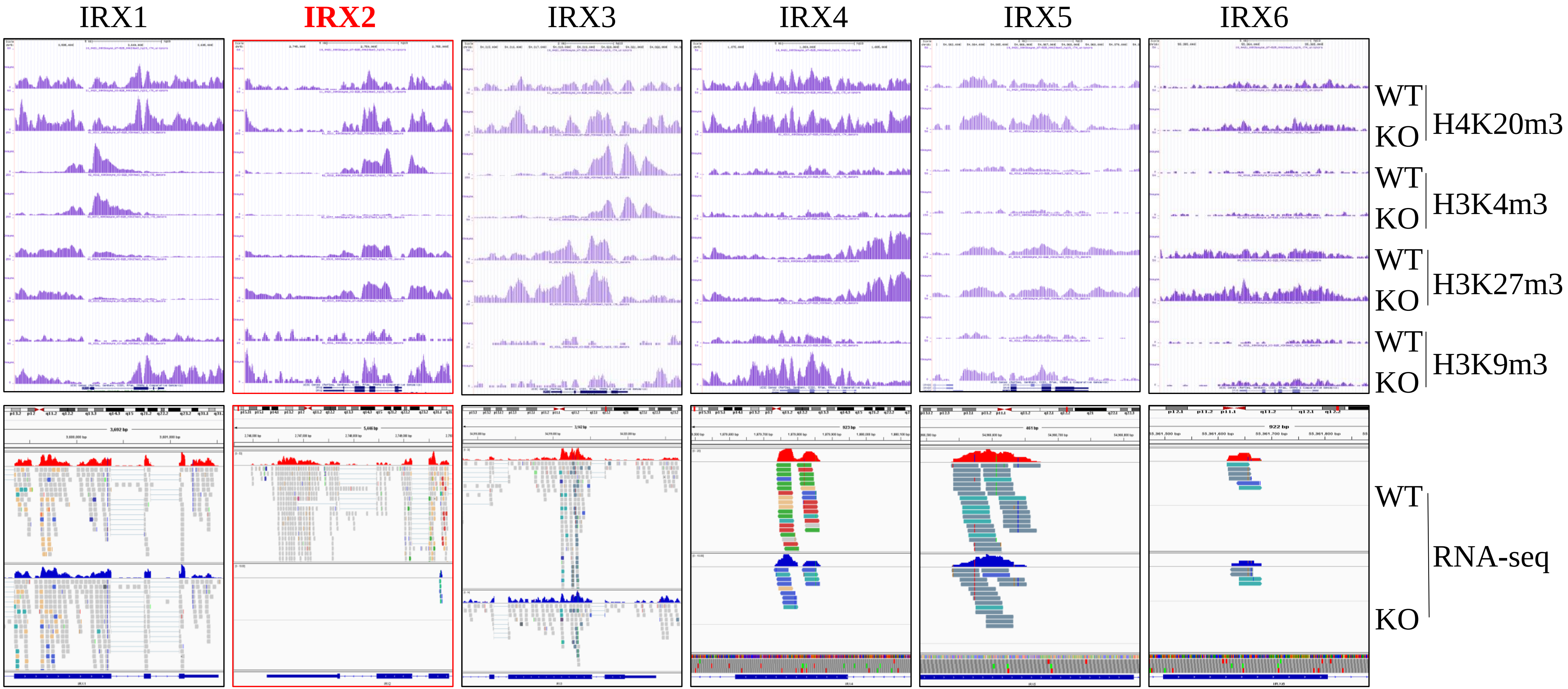
IRX1
IRX2
IRX3
IRX4
IRX5
IRX6
WT
KO
H4K20m3
WT
KO
H3K4m3
WT
KO
H3K27m3
WT
KO
H3K9m3
WT
KO
RNA-seq

Supplement figure legends:

sFig. 1. Establishment of the CRISPR-Cas9-mdig knockout cell lines. **A.** Targeting sequence of the sgRNA used in CRISPR-Cas9 vector for mdig knockout in BEAS-2B cells and MDA-MB-231 cells. Bottom panels show mdig knockout in the screened cell colonies of 2, 3, 4, 5, 6, and 8, but not the colonies of 1 and 7 of the BEAS-2B cells. **B.** RNA-seq diagram shows effective targeting of the CRISPR-Cas9 sgRNA at the exon 2 region of mdig gene in the mdig knockout cells (KO, marked with a red box), but not the cells without successful knockout of mdig (WT). **C.** Profiling of the histone H3 methylation by Westernblotting with antibodies against the mono-, di- and trimethylation of H3K4, H3K9, H3K27, and H3K36, and antibodies against histone H3 and GAPDH, respectively.

sFig. 2. ChIP-seq of H3R8me2a. Left panel: average plots of the merged peak regions of H3R8me2a between WT and mdig KO cells. Right panel: Heatmaps of the merged peak of H3R8me2a of the WT and mdig KO cells.

sFig. 3. Gene pathway analyses of the genes enriched with the repressive histone methylation markers in the mdig KO cells. An online program, g:Profiler was used for analysis of biological function (A, BP) and molecular function (B, MF), respectively.

sFig. 4. Histone methylation profiles of the key genes contributing to cell growth and stemness of the normal or cancer stem cells.

sFig. 5. RNA-seq spectrum of the genes listed in sFig. 3.

sFig. 6. RNA-seq spectrums of the indicated chemokines as detected in WT and mdig KO cells, respectively.

sFig. 7. Histone methylation profiles of the IRX gene family members and their RNA-seq expression between WT and mdig KO cells. No H3K4me3 peaks were detected for IRX4, IRX5 and IRX6, which correlates with their overall lower expression in RNA-seq in both WT and KO cells. IRX1, IRX2 and IRX3 showed significant enrichment of H3K9me3 and decrease of H3K4me3 in the KO cells. However, only IRX2 exhibited complete loss of H3K4me3 and RNA-seq expression in the KO cells.

AURKC [1]	-2.6
BICC1 [2]	-7.1
CLEC11A [3]	-10
CRISPLD1 [4]	-20
FGFR2 [5]	-2.4
FLRT3 [6]	-20
HLF [7]	-2.1
HOXA7 [8]	-10
ISL1 [9]	-2
KIT [10]	-4
MSC [11]	-20
PCDH7 [12]	-20
PDGFB [13]	-3
PDGFRA [14]	-2.2
RIPPLY2 [15]	-10
S1PR1 [16]	-3.8
SAMD11 [17,18]	-2.3
SOX4 [19]	-3.6
SPOCK1 [20]	-7.8
SPOCK3 [21]	-3.5
WNT5A [22]	-20
WNT7B [23]	-2.6
WNT9A [24]	-2.2

1 Quartuccio, S. M. & Schindler, K. Functions of Aurora kinase C in meiosis and cancer. *Front Cell Dev Biol* **3**, 50 (2015).
2 Lemaire, L. A. *et al.* Bicaudal C1 promotes pancreatic NEUROG3+ endocrine progenitor differentiation and ductal morphogenesis. *Development* **142**, 858-870 (2015).
3 Yue, R., Shen, B. & Morrison, S. J. Clec11a/osteolectin is an osteogenic growth factor that promotes the maintenance of the adult skeleton. *Elife* **5** (2016).
4 Holmfeldt, P. *et al.* Functional screen identifies regulators of murine hematopoietic stem cell repopulation. *J Exp Med* **213**, 433-449 (2016).
5 Coutu, D. L. & Galipeau, J. Roles of FGF signaling in stem cell self-renewal, senescence and aging. *Aging (Albany NY)* **3**, 920-933 (2011).
6 Muller, P. S. *et al.* The fibronectin leucine-rich repeat transmembrane protein Flrt2 is required in the epicardium to promote heart morphogenesis. *Development* **138**, 1297-1308 (2011).

7 Komorowska, K. *et al.* Hepatic Leukemia Factor Maintains Quiescence of Hematopoietic Stem Cells and Protects the Stem Cell Pool during Regeneration. *Cell Rep* **21**, 3514-3523 (2017).
8 Wheadon, H. *et al.* Differential Hox expression in murine embryonic stem cell models of normal and malignant hematopoiesis. *Stem Cells Dev* **20**, 1465-1476 (2011).
9 Bartulos, O. *et al.* ISL1 cardiovascular progenitor cells for cardiac repair after myocardial infarction. *JCI Insight* **1** (2016).
10 Ross, R. A., Walton, J. D., Han, D., Guo, H. F. & Cheung, N. K. A distinct gene expression signature characterizes human neuroblastoma cancer stem cells. *Stem Cell Res* **15**, 419-426 (2015).
11 Hishikawa, K. *et al.* Musculin/MyoR is expressed in kidney side population cells and can regulate their function. *J Cell Biol* **169**, 921-928 (2005).
12 Soady, K. J. *et al.* Mouse mammary stem cells express prognostic markers for triple-negative breast cancer. *Breast Cancer Res* **17**, 31 (2015).
13 Chen, W. *et al.* PDGFB-based stem cell gene therapy increases bone strength in the mouse. *Proc Natl Acad Sci U S A* **112**, E3893-3900 (2015).
14 Farahani, R. M. & Xaymardan, M. Platelet-Derived Growth Factor Receptor Alpha as a Marker of Mesenchymal Stem Cells in Development and Stem Cell Biology. *Stem Cells Int* **2015**, 362753 (2015).
15 Chan, T. *et al.* Ripply2 is essential for precise somite formation during mouse early development. *FEBS Lett* **581**, 2691-2696 (2007).
16 Li, M. *et al.* Co-stimulation of LPAR1 and S1PR1/3 increases the transplantation efficacy of human mesenchymal stem cells in drug-induced and alcoholic liver diseases. *Stem Cell Res Ther* **9**, 161 (2018).
17 Jin, G. *et al.* Identification and characterization of novel alternative splice variants of human SAMD11. *Gene* **530**, 215-221 (2013).
18 Ng, P. M. & Lufkin, T. Embryonic stem cells: protein interaction networks. *Biomol Concepts* **2**, 13-25 (2011).
19 Shen, H. *et al.* Sox4 Expression Confers Bladder Cancer Stem Cell Properties and Predicts for Poor Patient Outcome. *Int J Biol Sci* **11**, 1363-1375 (2015).
20 Shu, Y. J. *et al.* SPOCK1 as a potential cancer prognostic marker promotes the proliferation and metastasis of gallbladder cancer cells by activating the PI3K/AKT pathway. *Mol Cancer* **14**, 12 (2015).
21 Hawkins, F. *et al.* Prospective isolation of NKX2-1-expressing human lung progenitors derived from pluripotent stem cells. *J Clin Invest* **127**, 2277-2294 (2017).
22 Zhou, Y., Kipps, T. J. & Zhang, S. Wnt5a Signaling in Normal and Cancer Stem Cells. *Stem Cells Int* **2017**, 5295286 (2017).
23 Valkenburg, K. C., Graveel, C. R., Zylstra-Diegel, C. R., Zhong, Z. & Williams, B. O. Wnt/beta-catenin Signaling in Normal and Cancer Stem Cells. *Cancers (Basel)* **3**, 2050-2079 (2011).
24 Grainger, S. *et al.* Wnt9a Is Required for the Aortic Amplification of Nascent Hematopoietic Stem Cells. *Cell Rep* **17**, 1595-1606 (2016).
